# Comparing Long-term Mortality After Carotid Endarterectomy vs Carotid Stenting Using a Novel Instrumental Variable Method for Risk Adjustment in Observational Time-to-Event Data

**DOI:** 10.1001/jamanetworkopen.2018.1676

**Published:** 2018-09-07

**Authors:** Jesse A. Columbo, Pablo Martinez-Camblor, Todd A. MacKenzie, Douglas O. Staiger, Ravinder Kang, Philip P. Goodney, A. James O’Malley

**Affiliations:** 1 The Dartmouth Institute for Health Policy and Clinical Practice, Lebanon, New Hampshire; 2 Section of Vascular Surgery, Dartmouth-Hitchcock Medical Center, Lebanon, New Hampshire; 3 Department of Biomedical Data Science, Geisel School of Medicine, Lebanon, New Hampshire; 4 Department of Economics, Dartmouth College, Hanover, New Hampshire

## Abstract

**Importance:**

Choosing between competing treatment options is difficult for patients and clinicians when results from randomized and observational studies are discordant. Observational real-world studies yield more generalizable evidence for decision making than randomized clinical trials, but unmeasured confounding, especially in time-to-event analyses, can limit validity.

**Objectives:**

To compare long-term survival after carotid endarterectomy (CEA) and carotid artery stenting (CAS) in real-world practice using a novel instrumental variable method designed for time-to-event outcomes, and to compare the results with traditional risk-adjustment models used in observational research for survival analyses.

**Design, Setting, and Participants:**

A multicenter cohort study was performed. The Vascular Quality Initiative, an observational quality improvement registry, was used to compare long-term mortality after CEA vs CAS. The study included 86 017 patients who underwent CEA (n = 73 312) or CAS (n = 12 705) between January 1, 2003, and December 31, 2016. Patients were followed up for long-term mortality assessment by linking the registry data to Medicare claims. Medicare claims data were available through September 31, 2015.

**Exposure:**

Procedure type (CEA vs CAS).

**Main Outcomes and Measures:**

The hazard ratios (HRs) of all-cause mortality using unadjusted, adjusted, propensity-matched, and instrumental variable methods were examined. The instrumental variable was the proportion of CEA among the total carotid procedures (endarterectomy and stenting) performed at each hospital in the 12 months before each patient’s index operation and therefore varies over the study period.

**Results:**

Participants who underwent CEA had a mean (SD) age of 70.3 (9.4) years compared with 69.1 (10.4) years for CAS, and most were men (44 191 [60.4%] for CEA and 8117 [63.9%] for CAS). The observed 5-year mortality was 12.8% (95% CI, 12.5%-13.2%) for CEA and 17.0% (95% CI, 16.0%-18.1%) for CAS. The unadjusted HR of mortality for CEA vs CAS was 0.67 (95% CI, 0.64-0.71), and Cox-adjusted and propensity-matched HRs were similar (0.69; 95% CI, 0.65-0.74 and 0.71; 95% CI, 0.65-0.77, respectively). These findings are comparable with published observational studies of CEA vs CAS. However, the association between CEA and mortality was more modest when estimated by instrumental variable analysis (HR, 0.83; 95% CI, 0.70-0.98), a finding similar to data reported in randomized clinical trials.

**Conclusions and Relevance:**

The study found a survival advantage associated with CEA over CAS in unadjusted and Cox-adjusted analyses. However, this finding was more modest when using an instrumental variable method designed for time-to-event outcomes for risk adjustment. The instrumental variable-based results were more similar to findings from randomized clinical trials, suggesting this method may provide less biased estimates of time-dependent outcomes in observational analyses.

## Key Points

**Question:**

Can a novel instrumental variable method designed for time-dependent outcomes more accurately determine the relative long-term mortality after carotid endarterectomy vs carotid artery stenting?

**Findings:**

In this registry-based, multicenter cohort study of 86 017 patients, the hazard ratio of long-term mortality for carotid endarterectomy vs carotid artery stenting was 0.83 (95% CI, 0.70-0.98) using instrumental variable analysis, compared with 0.69 (95% CI, 0.65-0.74) using a traditional Cox regression model.

**Meaning:**

Results from this instrumental variable method show that the survival advantage conferred by carotid endarterectomy is more modest than suggested by traditional adjustment methods, aligning with results from randomized clinical trials.

## Teaser

This registry-based multicenter cohort study examines long-term survival after carotid endarterectomy (CEA) and carotid artery stenting (CAS) in real-world practice using a novel instrumental variable method designed for time-to-event outcomes compared with traditional risk-adjustment models used in observational studies for survival analyses.

## Introduction

Randomized clinical trials, which have internal validity and test efficacy under carefully designed study conditions, produce findings that are often widely accepted.^1,2,3,4^ When results of observational studies are concordant with randomized clinical trials, clear messages emerge for patients, clinicians, and payers to guide treatment decisions.^5,6,7,8^ Concordance of results is particularly important when assessing a long-term, time-to-event outcome such as mortality, as this suggests that the findings seen in randomized clinical trials will be durable in clinical practice.^9,10,11^

Treatment decisions are more difficult, however, when the results of randomized clinical trials and observational studies are discordant. For example, for patients and clinicians considering carotid endarterectomy (CEA) or carotid artery stenting (CAS), 2 competing treatments to prevent stroke from carotid artery stenosis, long-term survival after the procedure remains a matter of debate. While randomized clinical trials have shown no statistically significant difference in mortality between the 2 procedures, observational evidence suggests survival following endarterectomy is superior.^12,13,14,15,16,17^

Several potential explanations exist for this type of discordance.^18^ For example, treatment regimens and effects in randomized clinical trials may not reflect clinical practice, thereby limiting generalizability.^19,20^ This limitation of randomized clinical trials as well as their high cost and complexity make observational studies an attractive alternative. However, risk adjustment for confounding in observational data remains challenging. While methods such as Cox proportional hazards regression and propensity score matching have been developed to adjust observational time-to-event data for measured confounding, the possibility that unmeasured or even unmeasurable confounding persists in observational analyses is an important concern faced by patients and clinicians.^11,21,22,23,24^ Unmeasured confounding is of particular importance in patients with peripheral arterial disease considering invasive vs minimally invasive options, where surgeon selection bias and patient fitness for surgery have been shown to have an important association with clinical outcomes after aortic aneurysm repair.^25^ Selection bias and unmeasured confounding are likely to also occur in patients with carotid artery disease, where the decision to choose an invasive vs minimally invasive procedure is influenced by many factors.

Instrumental variable analysis is a procedure unique in its ability to account for unmeasured confounding, and this method has been applied to linear and logistic regression models to evaluate outcomes that are not time dependent.^26,27^ To date, adaptation of instrumental variable methods in areas of medicine such as cardiovascular disease that often examine time-to-event data using Cox regression as the standard analytic tool has been limited.^22,28,29,30,31^ We recently developed an instrumental variable procedure for use with the Cox model and have shown that it outperforms the traditional Cox model and 2-stage approaches that include the Cox model.^24,32^ We apply this procedure to adjust for suspected unmeasured confounding when comparing individuals’ long-term mortality between 2 competing treatments for carotid revascularization in a large observational data set.

## Methods

### Data Sources

Our analyses use data derived from the Vascular Quality Initiative registry, a national quality improvement registry that captures data on vascular procedures from more than 400 hospitals and practices across the United States and Canada.^33^ Patients and procedures entered in the registry were linked to the Medicare Denominator File for mortality assessment.^34,35^ This database includes patient-level information on baseline demographics, comorbid conditions, presenting neurologic symptoms, operative management, and mortality on patients who underwent CEA and CAS. Data from the Vascular Quality Initiative were available from January 1, 2003, to December 31, 2016. Medicare data were available until September 2015. All data were collected under the auspices of an Agency for Healthcare Research and Quality–designated Patient Safety Organization and were deidentified. Our study was approved by the Center for the Protection of Human Subjects at Dartmouth; a waiver of participant consent was obtained. This study was conducted in accordance with the Strengthening the Reporting of Observational Studies in Epidemiology (STROBE) reporting guideline.^36^

### Primary Exposure and Outcome

The primary exposure of interest was procedure type (CEA vs CAS). These procedures represent the most common methods of carotid revascularization in current practice.^37^ Patients receiving more than 1 procedure type in the same day were assigned to the first procedure they received. Patients receiving repeated revascularization procedures during follow-up were assigned to the index procedure.

The primary outcome was all-cause mortality. This was assessed for all patients in the registry using the Social Security Death Index. Patients were assessed from the time of their index procedure until death, and censored only at the end of their known follow-up period. Patients eligible for Medicare in the registry were linked to their respective Medicare claims file, analyzed through the end of September 2015. Successful linking was obtained in 92% and 91% of eligible patients who underwent CEA or CAS, respectively.

### Statistical Analysis

We performed a sensitivity analysis with results stratified based on the presence or absence of focal neurologic symptoms (symptomatic vs asymptomatic) at the time of presentation. We used clinical variables from the Vascular Quality Initiative to group patients as symptomatic or asymptomatic. We defined patients as symptomatic if they had a documented stroke, transient ischemic attack, or other ischemic neurologic symptoms at the time of hospitalization for their index procedure.

We reported unadjusted mortality as absolute and relative frequencies where appropriate. We calculated the hazard ratio (HR) of mortality for CEA vs CAS using 4 methods of estimation: unadjusted, Cox regression adjusted, propensity matched, and our instrumental variable procedure designed for time-to-event data. We applied these to the overall cohort, as well as in the sensitivity analysis based on the presence of focal neurologic symptoms at the time of revascularization. Statistical tests were 2-sided with *P* < .05 considered significant. All statistical analyses were performed with R, version 3.3.2 (R Foundation for Statistical Computing).

### Unadjusted and Adjusted Mortality

We calculated unadjusted mortality rates using Kaplan-Meier estimation. We then used Cox regression to estimate the HR of postoperative mortality for CEA vs CAS to account for observed confounding.^24,38^ Summary statistics for the confounding variables in the statistical models are noted in Table 1.

**Table 1.** Cohort Characteristics

Variable	All Patients (N = 86 017)		*P* Value	Propensity Matched (n = 24 680)		*P* Value
	CEA (n = 73 312)	CAS (n = 12 705)		CEA (n = 12 340)	CAS n = (12 340)	
Demographics						
Age, mean (SD), y	70.3 (9.4)	69.1 (10.4)	<.001	69.3 (9.7)	69.2 (10.4)	.44
Male, No. (%)	44 191 (60.4)	8117 (63.9)	<.001	7843 (63.6)	7867 (63.7)	.76
Race, No. (%)						
White	67 768 (92.4)	11 524 (90.7)	<.001	11 174 (90.6)	11 198 (90.8)	.65
Black	3099 (4.2)	694 (5.5)	<.001	681 (5.5)	672 (5.4)	.82
Other	2445 (3.4)	487 (3.8)	.005	485 (3.9)	470 (3.8)	.64
Clinical factors, No. (%)						
Elective	64 022 (87.3)	10 252 (80.7)	<.001	10 014 (81.2)	10 042 (81.4)	.66
Symptomatic	28 836 (39.3)	6863 (54.0)	<.001	6333 (51.3)	6519 (52.8)	.02
TIA or amaurosis	14 200 (19.4)	3106 (24.4)	<.001	2885 (23.4)	2970 (24.1)	.21
Stroke	14 636 (20.0)	3757 (29.6)	<.001	3448 (27.9)	3549 (28.8)	.16
Hypertension	65 128 (88.8)	11 292 (88.9)	.90	11 032 (89.4)	10 981 (89.0)	.31
Smoking history	55 476 (75.7)	9643 (75.9)	.59	9393 (76.1)	9361 (75.9)	.64
Positive stress test	5937 (8.1)	988 (7.8)	.23	977 (7.9)	973 (7.9)	.94
Coronary disease	20 643 (28.2)	4150 (32.7)	<.001	4153 (33.6)	4023 (32.6)	.08
Heart failure	7512 (10.2)	1883 (14.8)	<.001	1792 (14.5)	1782 (14.4)	.87
Diabetes	25 637 (35.0)	4595 (36.2)	<.001	4522 (36.6)	4451 (36.1)	.88
COPD	16 261 (22.2)	3233 (25.4)	<.001	3181 (25.8)	3107 (25.2)	.29
Renal insufficiency	4101 (5.6)	722 (5.7)	.70	722 (5.9)	702 (5.7)	.60
Hemodialysis	937 (1.3)	25 (0.2)	<.001	34 (0.3)	25 (0.2)	.30
Prior CEA	10 132 (13.8)	4132 (32.5)	<.001	3908 (31.2)	3803 (30.8)	.15
Medications						
Antiplatelet therapy, No. (%)						
Aspirin	60 744 (82.9)	10 877 (85.6)	<.001	10 363 (84.0)	10 545 (85.5)	.001
P2Y12 inhibitor	21 163 (28.9)	9646 (75.9)	<.001	9310 (75.4)	9281 (75.2)	.68
β-Blocker	41 759 (57.0)	7003 (55.1)	<.001	7005 (56.8)	6853 (55.5)	.04
Statin	58 588 (79.9)	10 120 (79.7)	.50	9833 (79.7)	9861 (79.9)	.67

Abbreviations: CAS, carotid artery stenting; CEA, carotid endarterectomy; COPD, chronic obstructive pulmonary disease; TIA, transient ischemic attack.

### Propensity-Matched Analysis

We created a propensity-matched cohort balanced in baseline covariates.^23,39^ Using the observed covariates in Table 1, we created a logistic regression model in which the dependent variable was the treatment exposure (CEA vs CAS). Next, we calculated the fitted probability of CEA, known as the propensity score, for each patient. We then matched patients undergoing CEA to those undergoing CAS. We compared mortality between patients who underwent CEA vs CAS in the matched cohort. To account for the censoring, we applied Cox regression to our propensity-matched cohort to estimate the HR of mortality for CEA vs CAS.

### Instrumental Variable Analysis

Our instrument was the proportion of CEA among the total number of carotid revascularization procedures (CEA and CAS) performed at each hospital in the 12 months prior to the index operation. We excluded hospitals not performing at least 10 revascularization procedures in the year prior to the index operation. In the presenting symptoms sensitivity analysis (patients presenting with focal neurologic symptoms vs not), we further excluded hospitals not performing at least 10 carotid revascularization procedures for each indication. For this reason, the number of patients included in the overall analysis slightly exceeds the total number of patients included in the sensitivity analysis.

The instrumental variable procedure identifies patients who would have undergone CEA at some institutions and CAS at others based on the value of the instrument alone and not on patient characteristics.^40^ If patients choose hospitals based on convenience, or at least based only on observed factors, then where a patient seeks treatment emulates a randomized encouragement design in which assignment to a hospital with a historical precedent for performing a high proportion of CEA randomly exposes the patient to a greater likelihood of undergoing CEA.^41^ The estimation of treatment effects using instrumental variables is well developed for linear or logistic regression models.^40,42,43^ However, Cox proportional hazard models examining time-to-event outcomes selects on survivors over the course of follow-up, which is problematic for standard methods of instrumental variable identification.^24,44,45,46^ Therefore, we used the new instrumental variable estimator for the Cox proportional hazards model to simultaneously deal with the problems of unmeasured confounding and censoring of the outcome.^32^ We used this new procedure to examine the HR for all-cause mortality after CEA vs CAS by including both the instrument and all known confounding variables described in Table 1. The mean (SD) value of the instrumental variable was 0.89 (0.12) for patients undergoing CEA and 0.65 (0.29) for patients undergoing CAS (*P* < .001). Further details on derivation of the instrumental variable and its distribution can be found in the eMethods and eFigure 1 in the Supplement.

### Instrument Assessment

We measured the strength of our instrument by determining if increasing levels of the instrument were associated with changing levels of the exposure.^47^ This is reported using the *F* statistic, for which a value greater than 10 traditionally indicates acceptable strength.^40^ The *F* statistic assesses the instrument’s ability to show association with the exposure received beyond the effect of any covariates that are adjusted for the survival model.

## Results

### Cohort Characteristics

We studied 86 017 patients who underwent carotid revascularization (CEA, n = 73 312; CAS, n = 12 705) from January 1, 2003, to December 31, 2016. Mean follow-up was 3.0 (SD, 2.4 years; range, <0.1-14.3 years; median [interquartile range] follow-up, 2.5 [1.3-4.0] years), yielding the equivalent of 259 700 person-years for analysis. Vital status was known for 75.0% of patients who were eligible (procedure date, 2011 or earlier). Compared with patients who underwent CEA, those who underwent CAS tended to be younger (mean [SD] age, 70.3 [9.4] vs 69.1 [10.4] years, respectively), were more likely to be male (44 191 [60.4%] vs 8117 [63.9%], respectively), and were more likey to have an urgent procedure (2453 [19.3%] vs 9290 [12.7%], respectively) (Table 1). More than 89% of patients were receiving some form of antiplatelet therapy, and more than 75% were receiving a statin. Characteristics of the sensitivity analysis cohorts (symptomatic and asymptomatic patients) were similar (eTables 1 and 2 in the Supplement).

There were several clinically meaningful differences between patients who underwent CEA and those who underwent CAS. Approximately one-third of patients undergoing CEA underwent the procedure because of focal neurologic symptoms, compared with more than half of patients treated with CAS. Patients who underwent CAS were also more likely to have several chronic comorbid conditions, including coronary artery disease, heart failure, and pulmonary disease. Patients who underwent CAS were also more likely to have previously undergone carotid surgery.

Given that several differences existed in the characteristics between patients treated with CEA and CAS, we created a propensity-matched cohort for analysis. The propensity-matched cohort consisted of 12 340 matched pairs of patients and was well balanced in baseline characteristics apart from a small difference in aspirin use (84.0% in the CEA group and 85.5% in the CAS group; *P* = .001), β-blocker prescription (56.8% in the CEA group and 55.5% in the CAS group; *P* = .04), and in the proportion of procedures performed for symptomatic stenosis (51.3% in the CEA group and 52.8% in the CAS group; *P* = .02). A graphical representation of the performance of the propensity score matching can be found in eFigure 2 in the Supplement.

### Unadjusted, Cox-Adjusted, and Propensity-Matched Mortality by Procedure Type

**Figure 1.**
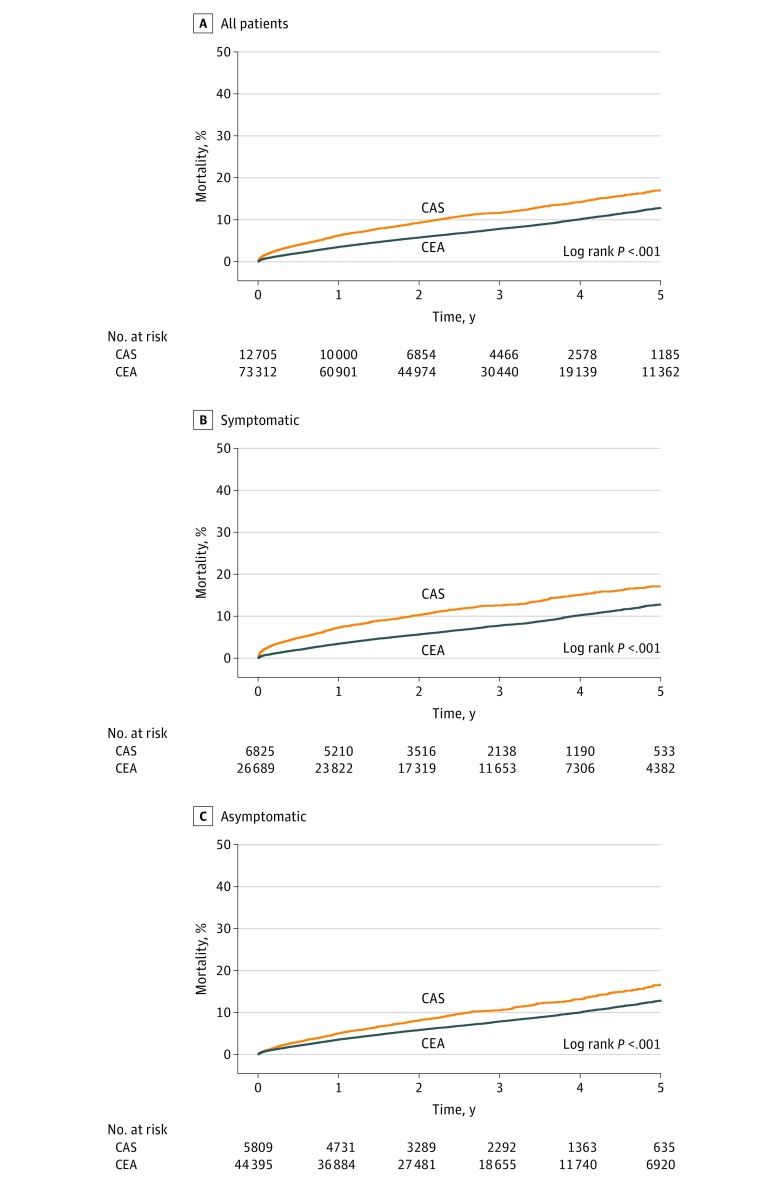
Kaplan-Meier Estimated Mortality, Overall and by Presenting Symptoms CAS indicates carotid artery stenting; CEA, carotid endarterectomy.

The unadjusted Kaplan-Meier estimate of all-cause mortality at 5 years for CEA was 12.8% (95% CI, 12.5%-13.2%) and for CAS was 17.0% (95% CI, 16.0%-18.1%; log rank, *P* < .001). At 10 years after the procedure, estimated mortality was 27.3% (95% CI, 26.3%-27.3%) for CEA and 27.4% (23.9%-30.7%) for CAS (log rank, *P* < .001; eFigure 3 in the Supplement). Sensitivity analysis by the presence of neurologic symptoms at the time of revascularization demonstrated similar findings (Figure 1).

**Figure 2.**
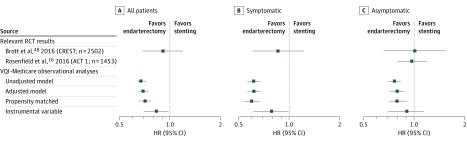
Hazard Ratios (HRs) of Mortality for Carotid Endarterectomy vs Carotid Stenting The CREST trial outcome represented is long-term stroke or periprocedural myocardial infarction, stroke, or death. ACT 1 indicates Asymptomatic Carotid Trial; CREST, Carotid Revascularization Endarterectomy vs Stenting Trial; RCT, randomized clinical trial; VQI, Vascular Quality Initiative.

The unadjusted HR of all-cause mortality for CEA vs CAS was 0.67 (95% CI, 0.64-0.71) (Table 2). A Cox proportional hazards model adjusting for differences in patient characteristics showed a similar association (HR, 0.69; 95% CI, 0.65-0.74), further suggesting that CEA was associated with a survival advantage. The propensity-matched cohort also revealed a survival advantage associated with CEA (HR, 0.71; 95% CI, 0.65-0.77). Sensitivity analysis by the presence of neurologic symptoms before carotid revascularization continued to show a statistically significant association (Table 2 and Figure 2).^16,48^

**Table 2.** Mortality HRs for Carotid Endarterectomy vs Carotid Stenting

Variable	No. of Mortality Events/Total No. of Patients^a^	HR (95% CI)			
		Crude	Adjusted	Propensity Matched	Instrumental Variable
Overall					
CEA	6600/73 312	0.67 (0.64-0.71)	0.69 (0.65-0.74)	0.71 (0.65-0.77)	0.83 (0.70-0.98)
CAS	1405/12 705	1 [Reference]	1 [Reference]	1 [Reference]	1 [Reference]
Symptomatic					
CEA	2559/28 689	0.61 (0.46-0.66)	0.61 (0.56-0.67)	0.59 (0.53-0.66)	0.78 (0.61-0.99)
CAS	786/6825	1 [Reference]	1 [Reference]	1 [Reference]	1 [Reference]
Asymptomatic					
CEA	4017/44 395	0.76 (0.70-0.83)	0.79 (0.72-0.87)	0.79 (0.71-0.90)	0.90 (0.70-1.14)
CAS	607/5809	1 [Reference]	1 [Reference]	1 [Reference]	1 [Reference]

Abbreviations: CAS, carotid artery stenting; CEA, carotid endarterectomy; HR, hazard ratio.

^a^ Event and total cohort numbers are different for the propensity-matched analysis.

### Instrumental Variable–Adjusted Mortality by Procedure Type

The instrument, each individual hospital’s 12-month prior proportion of CEA procedures, demonstrated a very strong association with the type of carotid procedure performed (*F* = 18 631). Applying our instrumental variable procedure to all-cause mortality revealed that patients selected for CEA had a more modest survival advantage (HR, 0.83; 95% CI, 0.70-0.98) than was suggested by results of our other analytic methods. These results are similar to the findings of published randomized clinical trials (Figure 2). Similar results were obtained by our instrumental variable approach in a sensitivity analyses stratified by presenting symptoms, although the association was more pronounced in those who were symptomatic. The HRs for those with symptoms changed by an absolute 17% to 19% between traditional statistical methods and our instrumental variable model, compared with an absolute change of 11% to 14% in those who were asymptomatic (Table 2).

## Discussion

In this observational study, unadjusted, adjusted, and propensity-matched models of long-term mortality all demonstrated that treatment with CEA was associated with a survival benefit relative to treatment with CAS. These results are comparable with published observational reports but conflict with randomized clinical trials, which suggest survival is similar following the 2 competing treatment options.^12,13,14,15,16,17,49,50^ Our instrumental variable method designed for risk adjustment of time-to-event data estimated a more modest association with long-term mortality, a finding consistent with the results of randomized clinical trials.^16^ These findings were robust to a sensitivity analysis by the presence of focal neurologic symptoms. This method, which accounts for both measured and unmeasured confounding in observational time-to-event analyses, represents an advance for investigators evaluating long-term outcomes, especially when considering clinical questions where randomized clinical trials are not possible or would be prohibitively expensive or when use of real-world evidence would be advantageous.^51^

Discordance between randomized clinical trials and observational studies is neither new nor uncommon.^52,53,54,55,56,57^ For example, differences in the efficacy of vitamin E and hormone replacement therapy for the prevention of heart disease as well as antioxidant therapy for cancer represent important examples where conflicting results from randomized clinical trials and observational studies have affected evidence-based treatment decisions.^52,53,54,55,56^ Meta-analyses examining the relative findings of randomized and observational studies suggest that observational studies tend to generate a larger treatment effect, and these differences may be further potentiated when assessing long-term outcomes such as mortality.^58,59,60,61,62,63,64^ However, this is not always the case; a recent Cochrane review estimated that treatment effects were similar between randomized clinical trials and observational studies (pooled ratio of odds ratios, 1.04; 95% CI, 0.89-1.21).^18^ These contradictory results highlight how challenging it can be for patients and clinicians to interpret observational study results, especially if the direction of bias cannot be foreseen.

In the example of discordance used in our analysis, patients with CAS, the Asymptomatic Carotid Trial (ACT 1) reported no statistically significant difference in 5-year mortality after CEA vs CAS,^16^ and the Carotid Revascularization Endarterectomy vs Stenting Trial (CREST) reported no statistically significant difference at 10 years in the composite outcome of long-term risk of stroke or perioperative myocardial infarction, stroke, or death.^48^ Despite these randomized clinical trials, several large observational studies have documented inferior outcomes for stenting overall, especially in subgroup analyses of symptomatic patients, which may bias physicians and patients away from choosing stenting as a procedural option.^12,13,14,15,50^ Our findings, which suggest that there is a modest association between survival and CEA, provide important granular detail to help inform this management decision.

The design and execution of a randomized clinical trial that provides true, unbiased estimates is a very difficult task, as there are several threats to both the validity and generalizability of their results. In some cases, clinical trial participants may not be representative of the target population.^18,19,20^ In others, heterogeneous treatment effects may impede the ability of physicians to parse out which patients may benefit most from an intervention.^18,19,20^ In addition, intention-to-treat estimators used in randomized clinical trials may be biased toward the null if noncompliance is considerable.^65^ These limitations to many contemporary randomized clinical trials highlight the utility of observational studies where real-world evidence can be used, provided that adequate adjustment for confounding can be performed.

An analytic technique capable of better risk adjustment for unmeasured confounding would improve the reliance that could be placed on results from observational studies. Such a technique would allow real-world observational data to more consistently reflect the true outcome of treatment independent of confounding. Our instrumental variable procedure was specifically designed to be used to analyze time-to-event outcomes.^32^ While determining a suitable instrument may be difficult in some settings, there appears to be few disadvantages in applying this procedure to observational questions with time-dependent outcomes such as mortality.^22,47,66,67^

While the findings of our instrumental variable analyses gave results that are similar to those in randomized clinical trials, this may not be the case when applied to other clinical scenarios. The importance of observational data is that it documents results from clinical practice, outside of the confines of randomized clinical trials. Observational studies often include a much broader patient population with treatment-effect heterogeneity than are found in randomized clinical trials.^20^ In these situations, results from instrumental variable analyses may be different than those found in randomized clinical trials and may better represent the results that can be expected when an intervention is incorporated into clinical practice. Application of instrumental variables to time-to-event data therefore represents an important step forward in the evaluation of interventions in contemporary practice.

### Limitations

Our study had limitations. First, it is not possible to truly know whether our instrumental variable balanced all unmeasured confounding. However, our sensitivity analyses by the presence of neurologic symptoms are reassuring. One would anticipate that unmeasured confounding would have a greater impact on the symptomatic analysis as patients in this subgroup are frequently sicker and thus are at higher risk for clinician selection bias to play a substantial role in the treatment decision. An instrument that accounts for unmeasurable confounding would change the effect size to a greater extent in these patients, and this was noted in our analyses. Second, we did not examine stroke-free survival as our primary outcome because of the heterogeneity in stroke assessment methods across the sites in our observational registry, an issue not encountered when examining survival as an outcome. Third, while 5-year vital status was known in 75.0% of patients who were eligible, many patients were not eligible for this assessment because of the date of their procedure (after 2011). Changes over time in both practice patterns and procedural competency may have an impact on the HR of mortality between the 2 procedures. However, findings remained consistent among patients who had their operations in earlier years where the longest follow-up was possible. Therefore, we feel that our estimates reported herein are an accurate reflection of long-term mortality after CEA vs CAS. In addition, instrumental variables must satisfy three conditions: first, they must be associated with the treatment exposure; second, an instrument must have no relationship to the outcome except through the effect on the exposure; and third, there must be no variables that affect both the instrument and the outcome.^68^ Our *F* statistic demonstrated that our instrument was strongly associated with the treatment exposure, thereby satisfying the first condition. It is not possible to prove whether an instrument is unrelated to an outcome. However, we required that a center perform at least 10 CEA or CAS procedures in the prior year to have patients included in the instrumental variable analysis to limit the possibility that proportion of procedures performed could be related to postoperative mortality.^69,70^

## Conclusions

Using a novel instrumental variable method designed for time-to-event data, we found only a modest difference in long-term mortality after CEA vs CAS, a result that is comparable with recent randomized clinical trials. These similarities provide evidence that results from our instrumental variable procedure are more closely aligned with the true relative long-term mortality between the 2 revascularization procedures than incumbent methods for analyzing observational data. This method, which allows instruments to be used for risk adjustment with the widely used Cox regression model, may improve the validity of results for time-dependent outcomes for clinical questions where randomized clinical trials are not possible or would be prohibitively expensive or when use of real-world evidence would be advantageous.

## Notes

Supplement. **eMethods.** Instrumental Variable Derivation **eFigure 1.** Distribution of the Instrumental Variable **eFigure 2.** Propensity Score Performance **eFigure 3.** Kaplan-Meier Estimated Mortality Overall and by Presenting Symptoms: 10-Years **eTable 1.** Characteristics of the Sub-Analysis Cohort: Symptomatic (n=35 514) **eTable 2.** Characteristics of the Sub-Analysis Cohort: Asymptomatic (n=50 204)
