# Error in Title and Figure

**DOI:** 10.1001/jamanetworkopen.2018.4055

**Published:** 2018-10-12

In the Original Investigation originally published with title, “Comparing Long-term Mortality After Carotid Endarterectomy vs Carotid Stenting Using a Novel Instrumental Variable Method for Risk Adjustment in Observational Time: Time-to-Event Data,”^1^ published September 7, 2018, there was an error in the title. The title should have appeared as “Comparing Long-term Mortality After Carotid Endarterectomy vs Carotid Stenting Using a Novel Instrumental Variable Method for Risk Adjustment in Observational Time-to-Event Data.” There was also an error in Figure 2. The data markers for “VQI-Medicare observational analyses” should have been shifted down by a row to correspond to their respective models. This article has been corrected.^1^
