# Supplementary material for: Comparing Long-term Mortality After Carotid Endarterectomy vs Carotid Stenting Using a Novel Instrumental Variable Method for Risk Adjustment in Observational Time-to-Event Data
Source: JAMA Netw Open. 2018 Sep 7;1(5):e181676. doi: 10.1001/jamanetworkopen.2018.1676 (PMC6324509; doi:10.1001/jamanetworkopen.2018.1676)
Supplement: Supplement. — eMethods. Instrumental Variable Derivation eFigure 1. Distribution of the Instrumental Variable eFigure 2. Propensity Score Performance eFigure 3. Kaplan-Meier Estimated Mortality Overall and by Presenting Symptoms: 10-Years eTable 1. Characteristics of the Sub-Analysis Cohort: Symptomatic (n=35 514) eTable 2. Characteristics of the Sub-Analysis Cohort: Asymptomatic (n=50 204) [file jamanetwopen-1-e181676-s001.pdf]

Supplementary Online Content

Columbo JA, Martinez-Camblor P, MacKenzie TA, et al. Comparing long-term mortality after carotid endarterectomy vs carotid stenting using a novel instrumental variable method for risk adjustment in observational time-to-event data. *JAMA Netw Open*. 2018;1(5):e181676. doi:10.1001/jamanetworkopen.2018.1676

eMethods. Instrumental Variable Derivation
eFigure 1. Distribution of the Instrumental Variable
eFigure 2. Propensity Score Performance
eFigure 3. Kaplan-Meier Estimated Mortality Overall and by Presenting Symptoms: 10-Years
eTable 1. Characteristics of the Sub-Analysis Cohort: Symptomatic (n=35 514)
eTable 2. Characteristics of the Sub-Analysis Cohort: Asymptomatic (n=50 204)

This supplementary material has been provided by the authors to give readers additional information about their work.

© 2018 Columbo JA et al. *JAMA Network Open*.

eMethods. Instrumental Variable Derivation

We assume that the risk model has the form:

$$\lambda(t|X,Z,U) = \lambda_0(t)\mathrm{ex}p(\beta_X \cdot X + \beta_Z \cdot Z + \beta_U \cdot U)$$

where X is the treatment, Z is a vector of measured confounders (see Table 1) and U is a potential unmeasured confounder. The target of inference is $\exp(\beta_X)$, the impact (in terms of a hazard ratio) of the treatment. In addition, we assume that the treatment assignment model has the form:

$$X = \alpha_0 + \alpha_{\mathrm{W}} \cdot \mathrm{W} + \alpha_{\mathrm{Z}} \cdot \mathrm{Z} + \alpha_{\mathrm{U}} \cdot \mathrm{U} + \epsilon$$

where W is the instrumental variable (IV) and ϵ is random error or white-noise. In our study, W is computed as the proportion of carotid endarterectomy (CEA) procedures out of the total CEA and carotid artery stenting (CAS) procedures performed over the past 12-months at the hospital where the patient underwent their procedure. Thus,

$$W = \frac{nCEA}{nCEA + nCAS},$$

where $nCEA$ and $nCAS$ denote the number of CEA and CAS procedures performed at the hospital in the 12-months prior to a given patient's procedure. Because the IV is patient-specific, it has the appealing property of varying within a hospital across time and so can exploit variation in a hospital's relative preference for CEA and CAS over time. To help validate the IV, the denominator of W is included as a measured confounder in the Cox model.

The two-stage residual inclusion frailty (2SRI-F) procedure estimates the treatment received by a patient via

$$\hat{X} = \widehat{\alpha_0} + \widehat{\alpha_W} \cdot W + \widehat{\alpha_Z} \cdot Z.$$

From this, we estimate the residuals, avoiding the estimation errors as

$$\widehat{\mathrm{R}} = \widehat{\mathrm{X}} - \mathrm{X} = \alpha_{\mathrm{U}} \cdot \mathrm{U} + \epsilon.$$

© 2018 Columbo JA et al. *JAMA Network Open*.

Finally, we add $\widehat{R}$ as a covariate in the Cox model and account for the white noise from the estimated first-stage equation, the quantity ϵ, by also adding an individual (gaussian) frailty term.

Assuming that all of the data are contained in a frame called DATA, the corresponding R code has the general form:

```
# FIRST STAGE. MODEL ASSIGNMENT ESTIMATION
```

- RES<- lm(X~ W + age + gender + race + etnia + […], data=DATA)$residuals

```
# SECOND STAGE. COX REGRESSION MODEL
```

- TSRF<- coxph(Surv(time,exitus)~ X + age + gender + race + etnia + […] + RES + frailty(1:nrow(DATA),dist="gaussian"), data=DATA)

© 2018 Columbo JA et al. *JAMA Network Open*.

eFigure 1. Distribution of the Instrumental Variable.

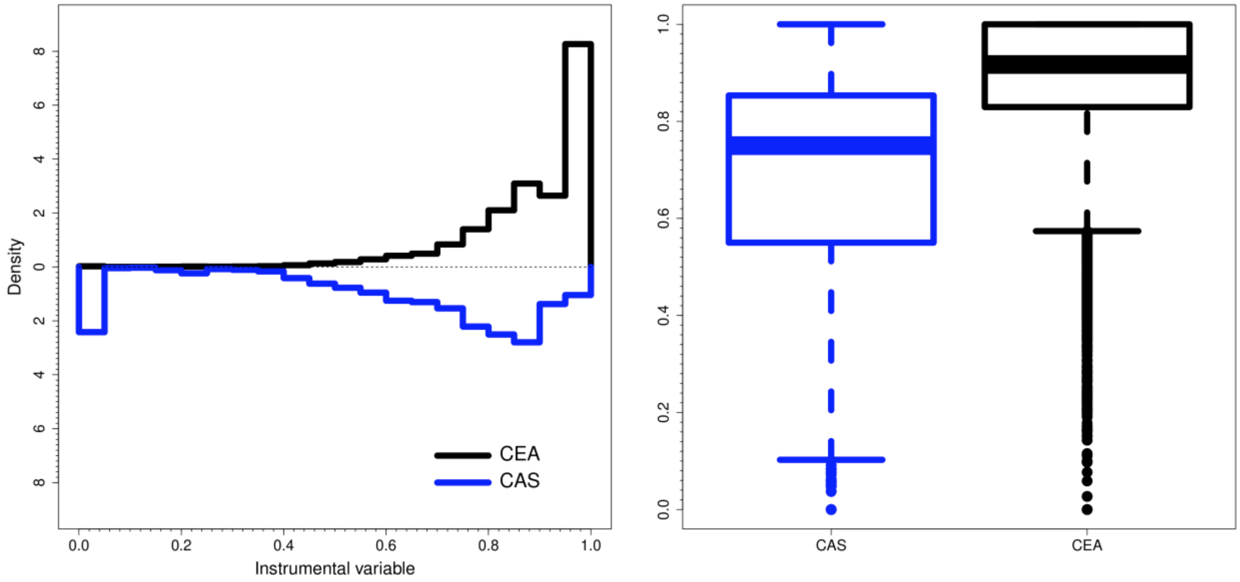

CEA = Carotid Endarterectomy; CAS = Carotid Artery Stenting.

© 2018 Columbo JA et al. *JAMA Network Open*.

eFigure 2. Propensity Score Performance

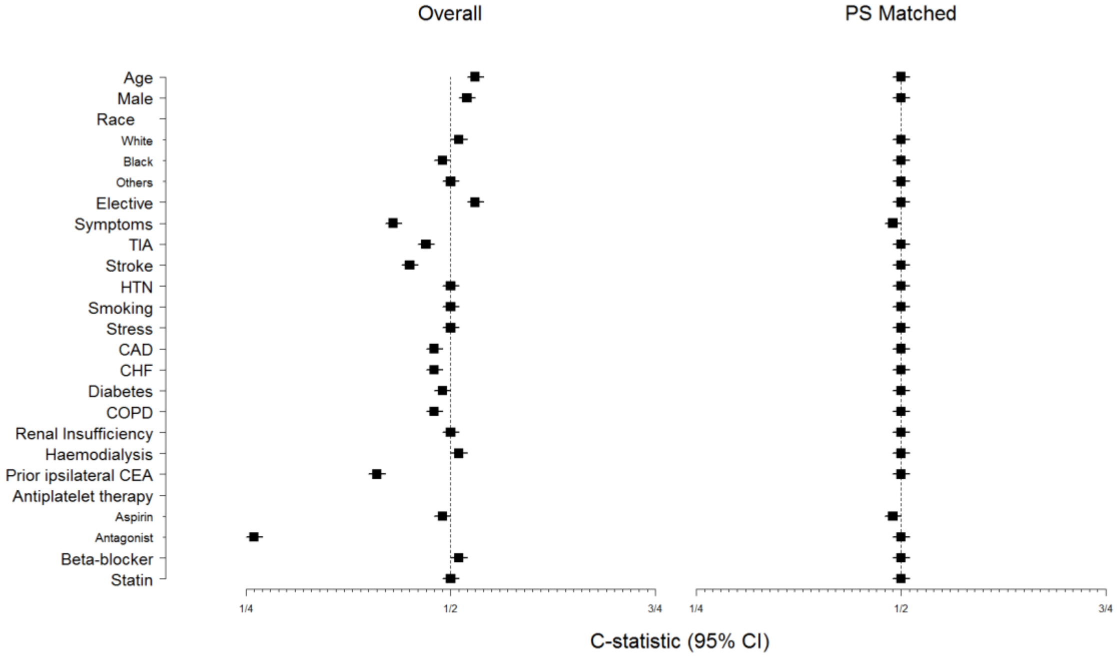

We used the traditional plot of the extent of the imbalance between the pre and post matched samples as a means to illustrate the performance of the propensity score matching procedure.

PS = propensity score; TIA = transient ischemic attack; HTN = hypertension; CAD = coronary artery disease; CHF = congestive heart failure; COPD = chronic obstructive pulmonary disease; CEA = carotid endarterectomy.

© 2018 Columbo JA et al. *JAMA Network Open*.

eFigure 3. Kaplan-Meier Estimated Mortality Overall and by Presenting Symptoms: 10-Years

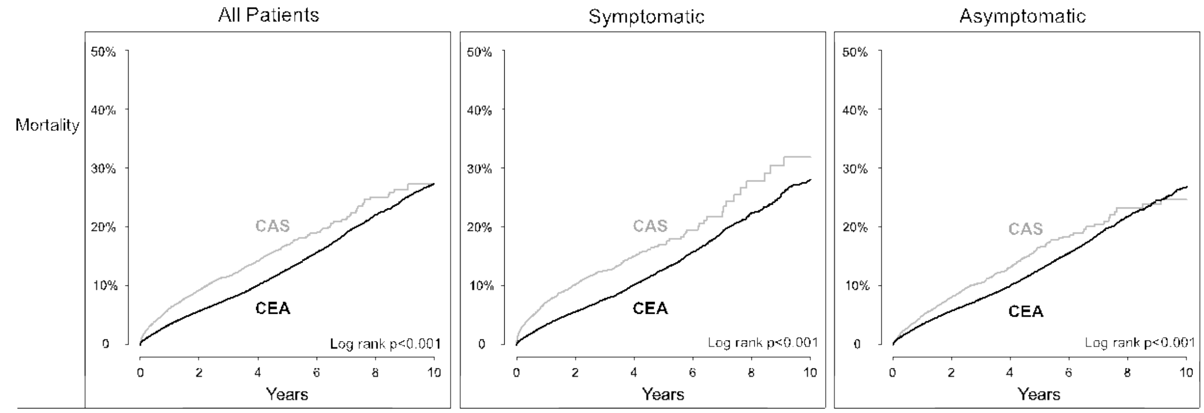

		All Patients 0	2	4	6	8	10	Symptomatic 0	2	4	6	8	10	Asymptomatic 0	2	4	6	8	10
CAS	At Risk:	12,705	6,854	2,578	487	182	99	6,825	3,516	1,190	192	60	37	5,809	3,289	1,363	295	122	62
	Mortality %:	-	9.3	14.2	19.0	25.0	27.4	-	10.3	15.1	19.6	27.8	31.9	-	8.1	13.2	18.4	23.2	24.6
CEA	At Risk:	73,312	44,974	19,139	6,935	3,688	2,064	26,689	17,319	7,306	2,661	1,410	787	44,395	27,481	11,740	4,249	2,272	1,272
	Mortality %:	-	5.8	10.1	15.7	20.1	27.3	-	5.7	10.3	15.8	22.4	28.0	-	5.9	10.0	15.6	21.8	26.9

Legend: CEA: carotid endarterectomy; CAS, carotid artery stenting.

© 2018 Columbo JA et al. *JAMA Network Open*.

eTable 1. Characteristics of the Sub-analysis Cohort: Symptomatic (n=35 514)*

Variable	All Patients		p	Propensity-Matched		p
	CEA n=28,689	CAS n=6,825		CEA n=6,418	CAS n=6,418	
**Demographics**						
Age mean (SD), years	70.2 (9.4)	68.7 (10.8)	<0.001	69.1 (9.8)	69.9 (10.7)	0.39
Male (%)	17,302 (60.3)	4,4169 (64.7)	<0.001	4,133 (64.4)	4,127 (64.3)	0.93
Race						
White	26,492 (92.3)	6,103 (89.4)	<0.001	5,765 (89.7)	5,757 (89.7)	0.83
Black	1,231 (4.3)	430 (6.3)	<0.001	375 (5.8)	389 (6.1)	0.63
Other	963 (3.4)	292 (4.3)	<0.001	278 (4.3)	272 (4.2)	0.83
**Clinical Factors**						
Elective	25,061 (87.3)	4,704 (70.2)	<0.001	4,805 (74.9)	4,725 (73.6)	0.11
Symptomatic	28,689 (100)	6,825 (100)	NA	6,418 (100)	6,418 (100)	NA
TIA or amaurosis	14,122 (49.2)	3,086 (45.2)	<0.001	2,980 (46.4)	2,947 (45.9)	0.57
Stroke	14,567 (50.8)	3,739 (54.8)	<0.001	3,438 (53.6)	3,471 (44.1)	0.57
Hypertension	24,487 (88.7)	6,045 (88.6)	0.72	5,723 (89.2)	5,691 (88.7)	0.38
Smoking History	21,662 (75.5)	5,128 (75.1)	0.53	4,933 (75.3)	4,834 (75.3)	1
Positive Stress Test	2,379 (8.3)	466 (6.8)	<0.001	472 (7.3)	446 (6.9)	0.39
Coronary Disease	8,086 (28.1)	2,071 (30.3)	<0.001	2004 (31.2)	1,962 (30.6)	0.43
Heart Failure	2,864 (10.0)	1,012 (14.8)	<0.001	905 (14.1)	916 (14.3)	0.8
Diabetes	10,044 (35.0)	2,491 (36.5)	0.02	2,350 (36.6)	2,334 (36.4)	0.34
COPD	6,199 (21.6)	1,714 (25.1)	<0.001	1,573 (24.5)	1,601 (24.9)	0.58
Renal Insufficiency	1,439 (5.0)	358 (5.2)	0.46	349 (5.4)	338 (5.4)	0.7
HD	332 (1.2)	14 (0.2)	<0.001	20 (0.3)	14 (0.2)	0.39
Prior CEA	4,013 (13.9)	1,935 (28.4)	<0.001	1,720 (26.8)	1,608 (25.1)	0.03
**Medications**						
Antiplatelet therapy						
Aspirin	23,851 (83.1)	5,751 (84.3)	0.03	5,279 (82.2)	5,377 (83.8)	0.02
P2y12 inhibitor	8,286 (28.9)	5,100 (74.7)	<0.001	4,824 (75.2)	4,693 (73.1)	0.009
Beta-blocker	16,339 (56.9)	3,544 (51.9)	<0.001	3,362 (52.4)	3,446 (53.7)	0.14
Statin	23,006 (80.2)	5,865 (78.6)	0.004	5,643 (77.5)	5,076 (77.7)	0.49

*Centers not performing at least 10 procedures on symptomatic patients in the year prior to analysis were excluded. For this reason the number of patients in the sub-analyses less slightly less than the number of patients in the combined analysis.

Legend: SD, standard deviation; CEA, carotid endarterectomy; CAS, carotid artery stenting; TIA, transient ischemic attack; NA, not applicable; COPD, chronic obstructive pulmonary disease; HD, hemodialysis.

© 2018 Columbo JA et al. *JAMA Network Open*.

eTable 2. Characteristics of the Sub-Analysis Cohort: Asymptomatic (n=50 204)*

Variable	All Patients		p	Propensity-Matched		p
	CEA n=44,395	CAS n=5,809		CEA n=5,737	CAS n=5,737	
**Demographics**						
Age mean (SD), years	70.3 (9.5)	69.5 (9.9)	<0.001	69.5 (9.5)	69.5 (9.9)	0.65
Male (%)	26,844 (60.5)	3,658 (63.0)	<0.001	3,603 (62.8)	3,632 (63.3)	0.59
Race						
White	41,064 (92.5)	5,356 (92.2)	0.42	5,311 (92.6)	5,290 (92.2)	0.48
Black	1,853 (4.2)	259 (4.5)	0.33	224 (3.9)	257 (4.5)	0.14
Other	1,473 (3.3)	194 (3.3)	0.96	202 (3.5)	190 (3.3)	0.57
**Clinical Factors**						
Elective	39,763 (87.3)	5,399 (92.9)	<0.001	5,328 (92.9)	5,329 (92.9)	1
Symptomatic	0 (0)	0 (0)	NA	0 (0)	0 (0)	NA
TIA or amaurosis	0 (0)	0 (0)	NA	0 (0)	0 (0)	NA
Stroke	0 (0)	0 (0)	NA	0 (0)	0 (0)	NA
Hypertension	30,464 (88.9)	5,181 (89.9)	0.51	5,116 (89.2)	5,122 (89.3)	0.88
Smoking History	33,641 (75.8)	4,464 (76.8)	0.08	4,449 (77.5)	4,414 (76.9)	0.45
Positive Stress Test	3,537 (8.0)	514 (8.8)	0.02	505 (8.8)	506 (8.8)	1
Coronary Disease	12,485 (28.1)	2,054 (35.4)	<0.001	2,017 (35.1)	2,022 (35.2)	0.94
Heart Failure	4,625 (10.4)	862 (14.8)	<0.001	783 (13.6)	828 (14.4)	0.24
Diabetes	15,512 (34.9)	2,684 (35.9)	0.17	2,060 (35.9)	2,063 (36.0)	0.97
COPD	10,015 (22.6)	1,501 (25.8)	<0.001	1,460 (25.4)	1,474 (25.7)	0.71
Renal Insufficiency	2,651 (6.0)	360 (6.2)	0.54	351 (6.1)	352 (6.1)	1
HD	599 (1.3)	11 (0.2)	<0.001	10 (0.2)	11 (0.2)	1
Prior CEA	6,098 (13.7)	2,178 (37.5)	<0.001	2,112 (36.8)	2,106 (36.7)	0.92
**Medications**						
Antiplatelet therapy						
Aspirin	36,702 (82.7)	5,063 (87.2)	<0.001	4,923 (85.8)	4,991 (87.0)	0.07
P2y12 inhibitor	12,800 (28.8)	4,491 (77.3)	<0.001	4,435 (77.3)	4,419 (77.0)	0.74
Beta-blocker	25,294 (57.0)	3,422 (58.9)	0.005	3,389 (59.1)	3,382 (58.9)	0.91
Statin	35,398 (79.7)	4,700 (80.9)	0.04	4,657 (81.2)	4,657 (81.2)	1

*Centers not performing at least 10 procedures on symptomatic patients in the year prior to analysis were excluded. For this reason the number of patients in the sub-analyses less slightly less than the number of patients in the combined analysis.

Legend: SD, standard deviation; CEA, carotid endarterectomy; CAS, carotid artery stenting; TIA, transient ischemic attack; NA, not applicable; COPD, chronic obstructive pulmonary disease; HD, hemodialysis.

© 2018 Columbo JA et al. *JAMA Network Open.*
